# Precision Enology Strategies to Enhance the Quality of Red Wine Color: The Synergistic Effect of pH and Selected Exogenous Grape Seed Tannins

**DOI:** 10.3390/foods15122161

**Published:** 2026-06-15

**Authors:** Arianna Ricci, Cristian Galaz Torres, Giuseppina Paola Parpinello, Antonio Pizzi, Andrea Versari

**Affiliations:** 1 Department of Agricultural and Food Sciences, Alma Mater Studiorum, University of Bologna, Piazza Gabriele Goidanich 60, 47521 Cesena, FC, Italy; andrea.versari@unibo.it; 2 Inter-Departmental Centre for Agri-Food Industrial Research, Alma Mater Studiorum, University of Bologna, via Quinto Bucci n. 336, 47521 Cesena, FC, Italy; 3 Departamento de Fruticultura y Enologìa, Facultad de Aronomìa y Sistemas Naturales, Pontificia Universidad Catòlica de Chile, Avenida Vicuña Mackenna 4860, Campus San Joaquín, Macul, Santiago 7820436, Chile; cristian.galaz@uc.cl; 4 Laboratoire d’Études et de Recherche sur le Matériau Bois (LERMAB), University of Lorraine, 27 Rue du Merle Blanc, BP 1041, 88051 Epinal, France; antonio.pizzi@univ-lorraine.fr; 5 Department of Physics, Faculty of Science, King Abdulaziz University, P.O. Box 80203, Jeddah 21589, Saudi Arabia

**Keywords:** red wine, wine color stability, oenological tannins, precision enology, climate change

## Abstract

Acidification and the application of exogenous tannins are well-established oenological practices designed to ensure wine stability and quality, playing a pivotal role to address the grape compositional imbalances associated with climate change. This study investigates precision enology techniques using a 2023 Sangiovese di Romagna, analyzing the interaction between pH modulation (3.2, 3.6, 3.8) and the addition of commercial grape seed tannins with varying medium degrees of polymerization (TanA: 3.1 mdp vs. TanB: 10.8 mdp). Following alcoholic fermentation, a full factorial design was implemented, including control batches (pH adjustment only). After a 40-day mild thermal treatment (T = 25 ± 1 °C) to simulate aging, results indicate that the high-mdp tannin (TanB) dominated color evolution regardless of pH, whereas the low-mdp tannin (TanA) effect was pH-dependent. Notably, a pH of 3.8 resulted in colloidal instability across all samples. The findings highlight the importance of customized protocols to mitigate climate-related challenges in winemaking.

## 1. Introduction

Color is a key organoleptic property of red wines, influencing quality perception and brand authority [1,2]. Notably, the initial visual impact dictates the overall assessment of aroma, taste, and mouthfeel [1,2]. During red winemaking, maceration is crucial for releasing anthocyanins from the skins; these pigments—together with numerous colorless compounds—undergo complex interactions, including copigmentation (Co), which enhances color intensity (hyperchromic effect) and shifts hues (bathochromic effect) [3], as well as the formation of polymeric pigments (PP) of varying molecular weight [4,5]. Due to the complexity of these reactions, defining the exact needs of the wine industry remains a critical endeavor. Furthermore, climate change has significantly altered grape composition at harvest, frequently leading to a misalignment between acid and phenolic maturity, resulting in musts with high pH and delayed phenolic development [6]. While red wine pH typically ranges from 2.8 to 4.0, even minor shifts significantly impact color quality, intensity, and stability [3,7,8]. Specifically, pH directly influences anthocyanin equilibria: the electrophilic flavylium ion prevails at low pH, whereas the colorless nucleophilic hydrate dominates at higher values [9]. pH also plays a pivotal role in copigmentation—essential for young wine color stability—which, however, becomes thermodynamically disadvantaged above pH 3.8 [3,10]. Concurrently, high pH values tend to accelerate oxidative processes by promoting the early synthesis of polymeric pigments, which lead to colloidal instability and color shades typical of aged wines [11,12,13]. Consequently, managing wine acidity in cellar practice, permitted within specific restrictions [14], has become crucial in modern enology for driving the physicochemical and colloidal stability of wine pigments [15].

Furthermore, exogenous oenological tannins can be added throughout the winemaking process to enhance color stability and quality, acting as antioxidants and cofactors that promote the reactivity of free anthocyanins (FA) toward more stable structures; their use is well-established in enology for their clarifying and stabilizing properties [16,17]. Oenological tannins from various botanical sources can be selected based on their specific reactivities: notably, condensed tannins (flavan-3-ols) with variable mean degrees of polymerization (mdp) from different grape sources (skin, seeds) are highly effective in color stabilization [16,18], with grape seed extracts being particularly suited for the scope [19,20]. However, selecting from commercial alternatives can be challenging due to the high compositional variability of commercial extracts, even when derived from the same source [16,21]. All these considered, we investigated the combined impact of intrinsic (pH) and extrinsic (commercial seed extracts with different mDP, ‘Tan’) factors on the color attributes of an iconic Italian wine, a Sangiovese 2023 from Emilia-Romagna, Italy. Accelerated aging experiments were conducted to identify optimal conditions across various technological scenarios. Despite limitations in replicating long-term wine aging with a mid-term thermal treatment, this approach yields a physicochemical evolution model that serves as a valuable foundation for future experiments.

## 2. Materials and Methods

### 2.1. Chemicals and Grape Seed Extracts

Chemicals including sodium chloride, sodium acetate, sodium dodecyl sulfate, triethanolamine, iron (III) chloride, BSA (≥98%), and benzyl mercaptan 99% (toluene-α-thiol) to prepare buffers and working solutions, along with the phosphorus red pigment (≥99.99%), were from Sigma (Sigma-Aldrich, St. Louis, MO, USA). Standard polyphenols (GA, PROT, SA, (+)-CAT, (-)-EPI, PRO B2, FERT, COUT, p-COUM, CA, RU, QUE-AGLC, QUE-GLC, Mv-3-*O*-glc) used for HPLC-DAD and spectrophotometric calibrations were reagent grade (≥98%) from Extrasynthese (Genay Cedex, France). The ultrapure water was from a MilliQ gradient system (Millipore Corporation, Billerica, MA, USA); methanol and acetonitrile for HPLC elution (≥99.9%) were from Merck (Merck Millipore, Darmstadt, Germany).

The grape seed extracts used in this experiment were commercial products (AEB Group, Brescia, Italy), both recommended for the post-fermentation addition to achieve optimum oxidative control and color evolution. After a preliminary screening of 20 commercial grape seed samples, the two tannins were chosen based on the differences in polymerized fraction and average degree of polymerization (see Table 1 and Table 2 and Section 3.1 and Section 4.1 for details). The extracts were labeled ‘TanA’ and ‘TanB’ and dissolved without any pre-treatment in distilled water (1 g/L) for the preliminary chemical characterization.

### 2.2. Vinification Protocol

A Sangiovese red wine vintage 2023 suitable for the production of ‘Romagna Sangiovese DOC’ was used in this experiment; it was industrially fermented and kindly supplied by the ‘Tenuta Nasano’ winery, located in the municipality of Riolo Terme, Emilia-Romagna, Italy. The 23 hectares of the vineyards, including the Sangiovese cultivar, have an altitude ranging from 150 to 230 m.a.s.l., and the soil is rich in clay, limestone, and potassium. The grapes (88 q) were mechanically harvested on 30 September 2023, and the vinification followed the standard winemaking protocol settled by the Company. In brief, the grapes were preserved from oxidation using tannins and mixed formulations of antioxidants (Tanicol One and No-ox uva, HTS Enologia, Marsala, Italy), prior to destemming–crushing; then, the must (84 hL) was inoculated with the yeast Lalvin ICV Black Pearl™ (Lallemand Inc. Italia, Verona, Italy) at a concentration of 10 g/hL. The fermentation-maceration process took place at a temperature of 22 ± 1 °C and lasted 7 days, followed by racking and pressing of the grape marc (SO_2_ addition: 0.2 g/ton). A new addition of potassium metabisulfite (0.2 g/ton) was provided to the second racking made after 5 days.

### 2.3. Proximate Analysis and Lab-Scale Experimental Design

Thirty liters of wine were delivered to the laboratory, allowed to complete the spontaneous malolactic fermentation, and analyzed for the basic enological parameters (Table 3) using the FTIR Bacchus 3 multiparametric analyzer (Steroglass Srl, Perugia, Italy) and the SO_2_ Glasschem Distiller (Blackheath, Cape Town, South Africa). After the analysis, the wine was divided into three batches (10 L each), and the pH was adjusted to 3.2, 3.6 or 3.8, respectively, using NaOH or HCl 1M; although the reference Regulation mandates the use of restricted chemical and physical procedures to regulate wine acidity [14], the use of inorganic acid and strong base was preferred for the laboratory scale simulation in order to achieve the desired pH values without causing a significant impact on the fixed acidity.

Every batch was further divided into three lots including the control (Ctrl) and two samples added with TanA and TanB, respectively: in order to work with a fixed Tan concentration, the dosage was standardized at the maximum value according to supplier’s recommendations (300 mg/L lyophilized powder), resulting in a bioactive content of approximately 70% for both extracts (details on composition in Section 3.1). Wines were bottled in 1 L glass (triplicate, total: 27 bottles), capped with crown caps under nitrogen flow and stored under mild thermal conditions (T = 25 ± 1 °C) for 40 days, to elicit the color development and simulate a mid-term wine evolution.

### 2.4. HPLC-DAD Methods

An Agilent Infinity 1290 LC (Agilent Technologies, Waldbronn, Germany) was used for the HPLC-DAD determinations. Polyphenol and anthocyanin monomers were detected and quantified according to previous HPLC-DAD methods described by the authors [23]; standards were dissolved in methanol/water mixtures in the range 0–50 mg/L to build calibrations (R^2^ > 0.98). To determine the medium degree of polymerization (mdp) of TanA and TanB, preparation was made before the injection using the thioacidolysis method of Vivas et al. [22], and mdp calculations were performed according to the same authors. Details on the HPLC-DAD method performance and calibration were provided as Supplementary Materials (Supplementary Table S1).

### 2.5. Matrix-Assisted Laser Desorption/Ionization Time-of-Flight Mass Spectrometry (MALDI-TOF MS)

Qualitative composition of the phenolic fraction was obtained using the MALDI-TOF MS method previously described by Ricci et al. [24]. The spectra were recorded using a Kratos compact MALDI Axima Performance TOF 2 (Shimadzu Biotech, Manchester, UK), with a nitrogen laser (337 nm), an ion gate for the selection of precursor ions, and a collision cell, using argon as the collision gas; NaCl was added in the sampling wells as the salt to enhance ion formation. The windows for separation of precursor ions were approximately 4 Da. Data were recorded in positive ion linear mode, applying the accumulation of 441 scans per spectrum, and the calibration of the linear modes was done over a mass range up to 2500 Da using phosphorus red pigment as a reference. The spectra were subjected to a raster analysis over the target, using the Maldi-MS software SampleStation software (v. 2016, Shimadzu Corp., Kyoto, Japan) for data treatment.

### 2.6. Spectrophotometric and CIELab Determinations

The TPC and PAs of TanA, TanB, and of the wine were measured using the AH assay [25]. Results were expressed in mg CE. The color fractions FA (%), Co (%) and PP (%), were obtained according to Boulton [3]. The LPP used to obtain the LPP/PP ratio was determined according to Harbertson et al. [26]. The CIELab chromatic coordinates L*, a*, b*, with illuminant D65 and 10° observer recommended by OIV [27], along with C* (Chroma) and H* (Hue angle) were obtained using the DNAPhone Smart Analysis technology (DNAPhone, Parma, Italy). The color stability was determined according to Alcalde-Eon et al. [2]: briefly, the wines after thermal aging were analyzed for the CIELab coordinates before and after cold storage (48 h, 4 °C). CIELab differences ΔE* > 3 were an indicator of a color change noticeable to the human eye and connected to remarkable colloidal instability phenomena (precipitation of colored matter).

### 2.7. Statistical Analysis

Mean differences in phenolic composition and color features of the experimental wines were evaluated by one- and two-way (with Tan and pH as factors) analyses of variance (ANOVA), using Tukey’s HSD as the post hoc test for the interaction effects. The agglomerative—hierarchical clustering with heatmap analysis (heatmap-AHC) were used to find natural configurations in the data according to the different experimental conditions, and to gain similarities between wines by grouping; due to the limited sample set being considered (total: 9 wines) and in order to remove any potential noise included in the data, Principal component analysis (PCA) was used as an unsupervised method for variables selection. All the analyses were carried out using XLSTAT version 2011.1.05 (Addinsoft, Anglesey, UK). All statistics were performed with significance at *p*-value ≤ 0.05.

## 3. Results

### 3.1. Compositional Profile of TanA, TanB, and Sangiovese Wine After Alcoholic Fermentation

#### 3.1.1. TanA and TanB

Analysis of TanA and TanB revealed similar TPC in both extracts (733 ± 6 mg/L CE for TanA and 724 ± 9 mg/L CE for TanB; Table 1). Conversely, the PAs content was consistently higher in TanB (305 ± 1 mg/L CE) than in TanA (188 ± 7 mg/L CE; Table 1). Acid-catalyzed depolymerization analysis yielded an mDP of 3.1 ± 0.6 for TanA (conversion yield 63.9 ± 5.4%), indicating short average chain length, and 10.8 ± 0.3 for TanB (72.5 ± 2.1% depolymerization), indicating long average chain length; the unreacted polymeric fraction was likely composed of PAs fragments lacking an acid-labile structure. Further insights into the composition were obtained from MALDI-TOF MS spectra (Supplementary Figures S1 and S2). Both TanA and TanB displayed typical grape seed PAs fragmentation patterns. Regarding monomers, distinct signals were observed: the main C peak (experimental: 291 Da), the sodium adduct (312 Da), and related fragments at 231 and 271 Da. The occurrence of GC-derived compounds was suggested by the main 303 Da peak paired with sodium adducts (experimental: 331 Da) and characteristic fragments at 258–220 Da. Signals with main peaks at 462 and 491 Da were assigned to the sodium adducts of CG and GCG, respectively, while the 152 Da fragment was attributed to galloyl units released during fragmentation. Supplementary Figure S1 shows the representative 100–500 Da pattern for TanA. The MALDI-TOF spectrum for this extract exhibited a limited signal for the polymeric fraction (*m*/*z* > 500 Da), with fragments linked to procyanidin B-type dimers: *m*/*z* 560 (dimer loss of -OH), *m*/*z* 554 (dimer loss of H_2_O), and *m*/*z* 530 (dimer loss of ethanol). A weak signal, likely a sodium adduct, was detected around *m*/*z* 600 (Supplementary Figure S1).

#### 3.1.2. Proximate Chemical Analyses of Sangiovese Wine After the Alcoholic Fermentation

Table 3 reports the Sangiovese composition and color at the end of the alcoholic fermentation. The TPC was 1949 ± 8 mg/L CE; it was primarily represented by flavonoids, i.e., FLAVAN (Σ = 782 ± 7 mg/L) and FLAVON (Σ = 35 ± 2 mg/L) compounds, and displayed comparable levels of HBA and HXCA (Σ = 52 ± 1 mg/L and Σ = 52 ± 0 mg/L, respectively). The PAs accounted for 37.0 ± 0.5% of the TPC (Table 3). The monomeric anthocyanins were mainly unacylated glucosides (Σ = 172 ± 3 mg/L MAE, Table 3), with large predominance of Mv-3-*O*-glc (78.5 ± 0.3% of the total monomeric anthocyanins determined by HPLC-DAD) followed by Pt-3-*O*-glc (9.9 ± 0.1%), Dp-3-*O*-glc (7.0 ± 0%), and Pn-3-*O*-glc (3.5 ± 0%); the Cy-3-*O*-glc was the minor monomeric anthocyanin (1.1 ± 0.3%). About the color fractions (3), FA (%) accounted for 63.0 ± 1.0% of the total color of the wine, whereas the extent of Co appeared limited at the end of fermentation (12.2 ± 1.1% of the total measured color, Table 3). As typical of early aged wines, the fraction of color due to PP (24.9 ± 0.2% of the total color, Table 3) was relatively low and primarily represented by small pigment polymers (LPP/PP: 17.8 ± 0.1%, Table 3).

### 3.2. Effect of pH and Tannins Addition Following Thermal Aging

#### 3.2.1. Phenolic Profile

The 40-day thermal aging induced distinct phenolic profiles based on pH levels and tannin concentration (Tan). To isolate the most discriminating chemical markers, a preliminary ANOVA (Table 4) followed by a PCA correlation matrix selection (squared cosines > 0.5, Supplementary Table S2) was applied. This statistical refinement led to the exclusion of non-discriminating variables (PROT, SA, (+)-CAT, FERT, p-COUM, CA, QUE-AGLC, and QUE-GLC) prior to heatmap-AHC analysis, optimizing the explained variance (PC1 increased from 49.22% to 55.00%; PC2 from 22.01% to 25.70%; Supplementary Figures S3 and S4) while preserving the original sample configuration.

**Figure 1:**
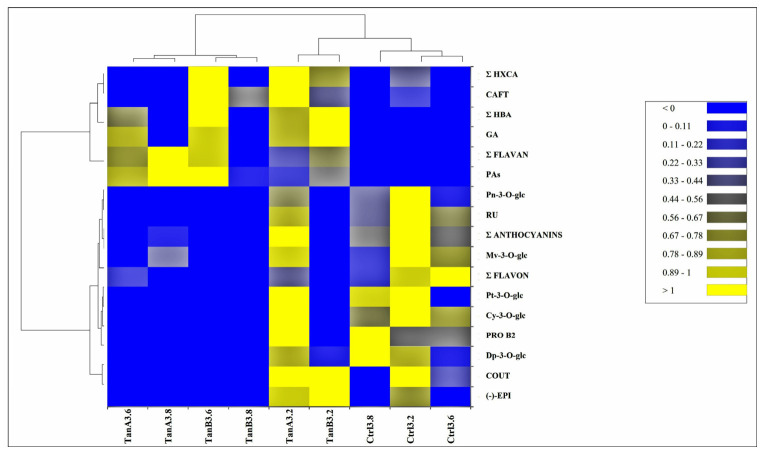
AHC—heatmap according to the phenolic composition of aged wines.

The resulting heatmap—AHC identified three groupings (Figure 1): Cluster 1 (TanA/TanB at pH 3.6–3.8), with marked depletion of free anthocyanins and phenolic monomers (i.e., RU, (-)-EPI); Cluster 2 (TanA/TanB at pH 3.2), with higher content of phenolic monomers, resembling the Ctrl wines (in particular the TanA 3.2 treatment); Cluster 3 (Ctrl wines) displaying the lowest overall levels of Σ HBA, Σ FLAVAN, PAs, and Σ HXCA (specifically CAFT, Table 4). However, among controls, the specific Ctrl3.2 maintained higher levels of anthocyanins, (-)-EPI, PRO B2, COUT, RU, and CAFT.

The evaluation of independent factors via one-way and two-way ANOVA (Table 4) revealed that pH variations governed specific phenolic subclasses without altering total anthocyanins. While total flavanols (Σ FLAVAN) remained unaffected by pH, the monomer (-)-EPI was strictly favored at pH 3.2. Furthermore, the experimental pH levels modulated the Σ HBA, Σ HXCA, and Σ FLAVON levels: lower GA was coupled with higher pH, SA had a significant depletion at pH 3.6, and both COUT (HXCA) and RU (FLAVON) peaked at pH 3.2, proving essential for pH-based discrimination.

Exogenous tannin addition (the independent ‘Tan’ factor) directly increased the total TPC and relative composition of polyphenols. Both TanB and TanA wines exhibited higher GA, likely derived from the native composition of the extracts (Table 1). Tannin addition triggered a sharp increase in total flavanols (Σ FLAVAN), driven by PAs (Table 4). The PRO B2 dimer followed an inverse trend, remaining significantly higher in Ctrl wines. Exogenous tannins also increased RU and QUE-GLC concentrations, whereas Σ HXCA did not reflect the slight increases observed for CAFT in TanA and TanB. The monomeric anthocyanin levels were generally dependent on treatments, with the exception of Dp-3-*O*-glc, which was not modified according to the experimental conditions. The synergistic pH × Tan interaction significantly impacted most phenolic compounds (Table 4), displaying a significant effect (*p* < 0.0001) across all phenolic subclasses. Within anthocyanins, this interaction was more critical for Cy-3-*O*-glc, Pt-3-*O*-glc, and Pt-3-*O*-glc (*p* < 0.0001) than for Mv-3-*O*-glc (*p* = 0.002) and Dp-3-*O*-glc (*p* = 0.003) levels, whereas individual compounds like (+)-CAT and selected HXCAs (FERT, p-COUM, CA) showed no significant interaction effects.

#### 3.2.2. Color Features

‘Tan’ and ‘pH’ acted as major drivers for color evolution during aging, as demonstrated by one-way ANOVA on key chromatic parameters (Table 5). The ‘Tan’ factor regulated the balance between free anthocyanins and copigmented structures: FA (%) peaked in control wines, whereas Co (%) increased following TanA or TanB addition.

The mean degree of polymerization (mdp) of the extracts was related to the development of polymeric pigments. Among the exogenous tannins, the higher-mdp extract (TanB, mdp 10.8) promoted a greater accumulation of PP (%) during aging without altering the LPP/PP ratio (Table 5). Conversely, the lower-mdp extract (TanA, mdp 3.1) generated Co (%) levels equivalent to TanB but failed to enhance polymeric pigments, yielding PP (%) values comparable to the control wines.

**Figure 2:**
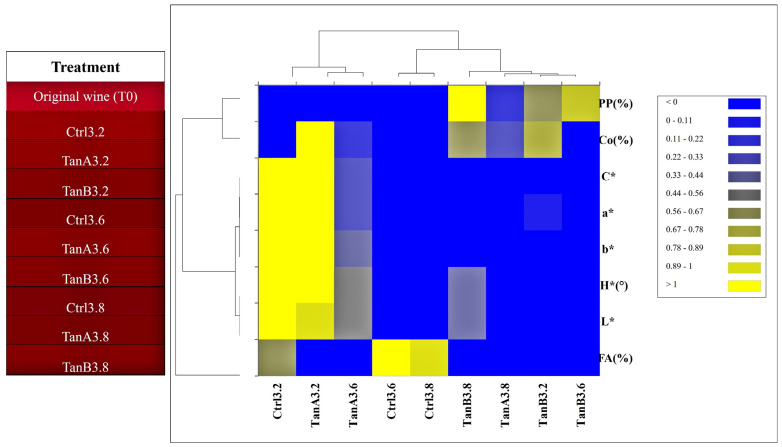
(**Right**): AHC—heatmap according to the colorimetric indexes of the aged wines and (**Left**): color rendering according to the CIELab coordinates.

pH had a significant impact on the mean Co (%). Maximum %Co values were observed at pH 3.2, driving an increase in mean a*, b*, and C* parameters (Table 5). Conversely, the pH 3.6 treatment, which is traditionally considered optimal for triggering copigmentation, yielded the lowest %Co, followed by pH 3.8. Individual factors did not significantly influence CIELab parameters, except for a*, b*, and C* at pH 3.2, but significant interaction effects were observed across all color traits. The exception was the LPP/PP ratio, which was not affected by different treatments and was excluded from the heatmap-AHC analysis (Figure 2).

The AHC analysis highlighted three distinct chromatic clusters (Figure 2): Cluster 1 (Ctrl3.2, TanA3.2, TanA3.6), with higher a*, b* indices and color saturation C*. Their hue (H°) shifted toward the red-yellow space with a pronounced tendency toward yellowish tones, while maintaining the highest lightness L*; Cluster 2 (TanB wines across all pH levels, and TanA3.8), with high PP (%) and relatively high Co (%). Within this group, TanB3.8 exhibited the highest PP (%) contribution and an increased H°, while TanB3.2 maximized the Co (%) effect; Cluster 3 (Ctrl3.6 and Ctrl3.8), with high FA (%) and reduced Co (%) and PP (%) contributions.

Color stability tests (Table 6) showed that pH control rather than tannin supplementation regulated the pigment stability during thermal stress. A significant mean color loss (ΔE* = 6.56) occurred in all pH 3.8 wines, indicating highly unstable pigments at higher pH, and the addition of grape seed extracts did not support the color stability.

## 4. Discussion

### 4.1. TanA and TanB Composition

The analysis of the two tannins (Tan) contributes to the debate on botanical extracts in enology, highlighting that identical sources can yield different, composition-dependent reactivity [16,21]. While TanA and TanB show similar TPC, their protein-reactive PAs levels differ (Table 1). Given the low monomeric content detected by HPLC-DAD (Table 1), the disparity between TPC and PAs points to a high dimer content with limited BSA reactivity. Moreover, MALDI-TOF confirms that TanA has a low polymerization degree (Table 1), with signals up to 600–627 Da tentatively assigned to sodium adducts of C-GC or C-C dimers (Supplementary Figure S1). TanB was categorized by the manufacturers as ‘rich in structured tannins’, and indeed the MALDI-TOF pattern of the extract reflects its structural complexity (Supplementary Figure S2). The signals of putative PAs with MW of 600–2500 Da relate to the occurrence of different B-type proanthocyanidins, primarily composed of C (EC), with occasional CG (EGC) repeat units [28]; the occurrence of GC-derived compounds, despite being uncommon in grape seed extracts [22], was previously reported [24,28,29]. TanB analysis revealed oligomers up to octamers with significant C (and occasionally GC) repeat units and galloylation (Table 2), supporting the high mdp calculated (Table 1). Besides tentative peak assignments, additional signals suggest greater structural complexity (e.g., GCG variants) or occasional -OH loss during PA fragmentation.

### 4.2. Compositional Profile of Sangiovese

Table 3 lists the physicochemical parameters of the Sangiovese wine measured soon after alcoholic fermentation. The phenolic composition matches existing literature, confirming that Sangiovese is relatively poor in anthocyanins compared with other cultivars, largely due to unstable dihydroxy structures (e.g., Dp-3-*O*-glc and Cy-3-*O*-glc) that decrease during winemaking [30,31]. In this study, stable methoxylated forms constituted a mean of 91.9% of total anthocyanins after alcoholic fermentation (Table 3), with a high prevalence of Mv-3-*O*-glc (78.5%), followed by Pt-3-*O*-glc (9.9%) and Pn-3-*O*-glc (3.5%). Mono- and di-hydroxylated compounds were limited, with Dp-3-*O*-glc representing 7.0% of the total monomers (Table 3). According to the literature [18,32] Co typically accounts for 30–50% of the total measured color in young red wines, dropping soon after fermentation (Table 3); the b* (30.37 ± 0.15, Table 3) and H* (28.07 ± 0.0°, Table 3) for the wine used in this experiment exhibited a slight deviation from the typical purple hues previously reported for a young red wine (b* 36.10 and H* 33.26° [27]) to a moderate contribution of yellow tones. This is consistent with previous reports by Boulton [3], which listed Sangiovese among the grapes with low cofactors, resulting in a limited copigmentation extent and more reddish color hues.

### 4.3. Simulated Aging Experiment

Accelerated aging tests are standard protocols in wine science to predict oxidative evolution compared with real-time aging [33,34]. Artificial aging techniques include heat, enzymes, hydrogen peroxide, or acetaldehyde addition [35]. We chose thermal aging as a mild, controlled method for this study to accelerate kinetic processes—such as oxidative formation of polymeric pigments (PP)—while facilitating the copigmentation onset, which is thermodynamically favored at lower temperatures [18]. To maintain the structure of the Co complexes, the temperature was set to 25 ± 1 °C, as higher temperatures decrease stability through solvent effects on the flavylium cation [36,37].

The analysis of the phenolic fraction reveals potentially competing reaction mechanisms during aging, highlighted by a systematic increase in GA in TanA and TanB wines, alongside a sharp depletion of GA across all pH 3.8 treatments (Table 1). This pH-dependent decline points to two coexisting pathways: (i) the formation of stable, complex copigments [38,39], or (ii) oxidative degradation [40,41]. Regarding the first mechanism, model systems evaluating GA-oenin (Mv-3-*O*-glc) copigmentation define GA as a weak copigment with low Gibbs free energy (ΔG0: −7.6 kJ/mol experimental [38]; −8.99 kJ/mol theoretical [39]). This binding affinity is weaker than that of other wine hydroxybenzoic acids (HBAs), such as syringic acid (SA) (ΔG0: −9.6 kJ/mol experimental [42]; −9.8 kJ/mol experimental [39]; −17.20 kJ/mol theoretical [39]), and significantly less effective than hydroxycinnamic acids (HXCAs) and flavan-3-ol cofactors (ΔG0 < 10 kJ/mol [38]). The SA concentration had a significant drop at pH 3.6 (Table 4), aligned with literature reporting SA as the primary cofactor among wine HBAs due to its di-ortho methylated structure, which optimizes stability at pH 3.6 [42]. However, because the absolute measured concentrations were extremely low (<0.5 mg/L) in our study, this specific drop likely holds negligible practical relevance for the overall chromatic evolution of wine. The pH-independent increase in total flavanols (ΣFLAVAN) in all tannin-supplemented wines (TanA and TanB) contrasted with a striking reverse trend for the dimeric procyanidin PRO B2, which was systematically lower in all Tan treatments compared with the Ctrls. According to the literature [43], the copigmentation extent (Co%) is dependent on specific pigment-to-copigment ratios, typically optimized between 1:20 and 1:40; given the characteristically low monomeric anthocyanin content of Sangiovese wines (see Section 4.2) and the high concentration of dimeric procyanidins in our TanA/B extracts (see Section 4.1), we hypothesize that PRO B2 was selectively consumed during aging to drive pigment evolution, causing a substantial decrease in its free form. Furthermore, the strong modulation of both PAs and PRO B2 levels by the synergistic pH × Tan interaction confirms their active role in early-stage copigmentation and subsequent polymeric pigment (PP) accumulation [44].

Tannin supplementation systematically increased CAFT and COUT levels, particularly in the TanB3.6 and TanB3.8 treatments compared with their respective controls (Table 4). This trend is possibly related to the antioxidant protection afforded by the added extracts. The literature presents conflicting views on how HXCAs mediate wine pigment formation, with a split between early involvement in pH-dependent copigmentation or a direct, pH-independent reaction yielding orange-hued pyranoanthocyanin-phenol structures [45,46]. Because the independent pH factor did not significantly impact HXCA levels in our study (Table 4), our data seem to support the second, pH-independent pathway. Regarding flavonols, QUE-GLC was highly sensitive to pH × Tan interactions, showing a consistently greater reduction in its free form upon TanB addition; a trend completely absent for the less soluble quercetin aglycone (QUE-AGLC) (Table 4). This selective reduction suggests enhanced flavonol–flavanol interactions supporting previous evidence of stable ternary copigmentation complexes formation [47,48]. This observed synergy between grape seed extracts and QUE-GLC might offer a practical color-fining solution for Sangiovese wines, which are frequently prone to stability issues due to high flavonol accumulation [49,50]. The anthocyanin monomers showed a clear reactivity hierarchy, where the pH × Tan synergy preferentially consumed Pt-3-*O*-glc, Pn-3-*O*-glc, and Cy-3-*O*-glc (Table 4). Specifically, the Cy-3-*O*-glc and Pt-3-*O*-glc levels were systematically lower at pH 3.6–3.8 and under TanB addition (Table 4). Conversely, Mv-3-*O*-glc remained stable regardless of pH but decreased significantly in TanB treatments, exhibiting a distinct pH × Tan interaction (Table 4). These varying degradation patterns confirm that different anthocyanins follow distinct, competing reaction pathways [51,52]. Furthermore, they highlight that stable wine pigments do not evolve uniformly from a single copigmentation model, allowing certain combinations to yield high hyperchromicity with minimal spectral shifts [51,52]. The dominant impact of TanB on wine color was confirmed by the heatmap-AHC, which grouped all TanB treatments into Cluster 2 based on color similarities despite varying pH levels (Figure 2). Cluster 2 was characterized by low FA (%), high PP (%), and similar CIELab profiles, including higher color intensity (lower L*) and bluer tones (lower b*). Within this cluster, TanB3.6 exhibited lower Co (%) than TanB3.2 and TanB3.8. Because copigmentation is traditionally favored at pH 3.6 [3,18], the occasional drop indicates that the TanB3.6 treatment experienced accelerated early color evolution toward highly stable, condensed polymeric forms.

Clustering revealed that TanA exhibited a more pronounced pH dependence than TanB. TanA3.8 grouped within Cluster 2, sharing distinct chromatic traits with the TanB wines. Conversely, TanA3.2 and TanA3.6 clustered with Ctrl3.2 in Cluster 1, characterized by higher Co (%), color saturation C*, and a more pronounced red-yellowish hue (higher H*, a*, and b* values). The different pigment reactivity may stem from the lower concentrations of QUE-GLC found in these samples; since QUE-GLC participates in copigmentation pathways that induce high hyperchromicity alongside restricted bathochromic (or even hypsochromic) shifts, it may produce red-yellowish tones. Furthermore, because TanA3.2 and TanA3.6 shared the highest free GA levels, their behavior points to underlying stabilization mechanisms. This aligns with independent studies [53,54] demonstrating that GA exerts a protective effect against thermally induced color fading, a phenomenon attributed to its optimal 4.37 Å stacking distance from the anthocyanin plane.

Regarding color stability, a noticeable difference after the cold test (ΔE* > 3.0) was observed in wines with a higher pH (3.8), whereas groups with a pH of 3.2 and 3.6 maintained ΔE* values below the threshold of human perception (Table 6). In the case study, the additions of TanA and TanB provided no stabilizing effects for higher pH; this finding warrants further research, as grape seed extracts are frequently evaluated as a viable approach to preventing color instabilities related to systematic pH increases in industrial wines [2,20,55,56].

We explored the role of pH adjustment and exogenous tannins on mid-term color evolution in Sangiovese wine. Because phenolic compounds react differently to anthocyanins under technological conditions, the type and dosage of exogenous extracts must be carefully managed alongside the wine’s native phenolic profile. While custom approaches should prioritize the grape’s specific composition, our findings regarding extract reactivity and the protective role of phenolic monomers offer generalizable insights. Notably, adjusting pH proved more effective for color and tannin stability than adding commercial tannins. Further studies are needed to understand colloidal stability and develop protective strategies under changing climatic conditions.

## 5. Conclusions

This study proposes a systematic approach to optimize red wine color stability, using Sangiovese to test the impact of pH and exogenous tannins. Findings indicate that the post-fermentative addition of high-mdp seed tannins (TanB) at a typically recommended dosage (30 g/hL) effectively stabilizes color in low-to-medium pH (3.2–3.6) wines. In contrast, at pH 3.8, seed extracts yielded no benefits on color stability, emphasizing the necessity of acidification. The study develops a methodological framework that accounts for varying wine compositions. This approach, when implemented within a Decision Support System (DSS), enables the industry to enhance sustainability through optimized interventions, including tailored acidification and the reduction in unnecessary adjuvants.

## Figures and Tables

**Table 1:** Polyphenolic composition of the commercial grape seed extracts used in the experiment (values are expressed per g dry weight of the extract). n.d.: not detected.

Polyphenolic Compounds	TanA	TanB
TPC—(mg CE/g dw)	733 ± 6	724 ± 9
PAs—(mg CE/g dw)	188 ± 7	305 ± 1
mdp	3.1 ± 0.6	10.8 ± 0.3
Thioacidolysis yield (% ^1^)	63.9 ± 5.4	72.5 ± 2.1
GA (mg/g dw)	5.9 ± 0	6.8 ± 0.4
SA (mg/g dw)	4.6 ± 0.7	2.5 ± 0.7
(+)-CAT (mg/g dw)	5.6 ± 0.1	6.5 ± 0.3
(-)-EPI (mg/g dw)	4.5 ± 0.2	5.4 ± 0.1
PRO B2(mg/g dw)	7.5 ± 0.5	3.5 ± 0.1
QUE-GLC (mg/g dw)	3.8 ± 0.3	n.d.
CA (mg/g dw)	2.0 ± 0.3	2.3 ± 0.2

^1^ Calculated according to [22].

**Table 2:** MALDI-TOF MS fragmentation of sample TanB (linear positive mode, ion gate: 400 Da) recorded in the range 600–2500 Da.

Observed [M^+^ + Na^+^]	Calculated [M^+^ + Na^+^]	Peak Assignment
629	629	2 GC dimer
939	932	3 GC trimer
1196	1190	3 C+ 1 GC tetramer; 1 C + 2 CG trimer (isobaric)
1205	1205	2 C + 2 GC tetramer
1545	1538	5 GC pentamer
1745	1751	6 C hexamer
1831	1826	1 C + 5 GC hexamer
1907	1907	5 C + 1 CG hexamer
2109	2114	2 C-5 GC heptamer
2194	2189	6 C + 1 CG heptamer
2347	2342	7 C + 1 GC octamer; 5 C + 2 CG heptamer (isobaric)
2367	2372	5 C + 3 GC octamer; 2 C + 4 CG hexamer (isobaric)
2406	2402	3 C + 5 GC octamer
2441	2447	8 GC octamer
2483	2485	7 C + 1 CG octamer

**Table 3:** Proximate chemical analysis of the Sangiovese red wine 2023 after alcoholic fermentation.

Wine Parameters	
Alcohol (%)	14.35 ± 0.03
Reducing sugar (g/L Glu)	3.55 ± 0.33
pH	3.65 ± 0.02
Total acidity (g/L TA)	6.19 ± 0.05
Volatile acidity (g/L AA)	0.34 ± 0.01
SO_2_, free (mg/L)	8.8 ± 0.4
SO_2_, total (mg/L)	28.0 ± 1.5
TPC (mg/L CE)	1949 ± 8
PAs (mg/L CE)	732 ± 5
GA (mg/L)	47.5 ± 0.5
PROT (mg/L)	3.5 ± 0.5
SA (mg/L)	0.6 ± 0.2
(+)-CAT (mg/L)	18.3 ± 1.3
(-)-EPI (mg/L)	11.6 ± 1.11
PRO B2 (mg/L)	20.2 ± 1.5
FERT (mg/L CAE)	2.9 ± 0.1
COUT (mg/L CAE)	9.6 ± 0.2
p-COUM (mg/L)	0.2 ± 0.0
CAFT (mg/L CAE)	37.9 ± 0.1
CA (mg/L)	1.4 ± 0.0
RU (mg/L)	3.2 ± 0.4
QUE-AGLC (mg/L)	5.6 ± 0.2
QUE-GLC (mg/L)	26.5 ± 1.1
Dp-3-*O*-glc (mg/L MAE)	12.0 ± 0.2
Cy-3-*O*-glc (mg/L MAE)	2.0 ± 0.6
Pt-3-*O*-glc (mg/L MAE)	17.0 ± 0.2
Pn-3-*O*-glc (mg/L MAE)	6.0 ± 0.1
Mv-3-*O*-glc (mg/L)	134.9 ± 1.5
L*	27.2 ± 0.3
a*	56.57 ± 0.05
b*	30.37 ± 0.15
C*	64.21 ± 0.11
H* (°)	28.07 ± 0.0
FA (%)	63.0 ± 1.0
Co (%)	12.2 ± 1.1
PP (%)	24.9 ± 0.2
LPP/PP (%)	17.8 ± 0.1

Legend: Glu: glucose equivalent; TA: tartaric acid equivalent; AA: acetic acid equivalent.

**Table 4:** Mean separation of polyphenol monomers (one-way and two-way ANOVA) of Sangiovese red wines obtained under different pH and Tan conditions, along with the interactive effect of both factors. Letters next to the values indicate groups based on Tukey HSD comparison tests. Signification codes: *** *p*-value < 0.001; ** *p*-value < 0.01; * *p*-value < 0.05; *p*-value < 0.1; n.s. not significant.

Two-Way ANOVA																								
pH		Tan			GA (mg/L)		PROT (mg/L)		SA (mg/L)			Σ HBA			(+)-CAT (mg/L)			(-)-EPI (mg/L)		PRO B2 (mg/L)		PAs (mg/L CE)		Σ FLAVAN (mg/L)
pH		pH3.2			54.74 ± 1.44 b		2.85 ± 0.17 a		0.45 ± 0.15 b			58.04 ± 1.27 b			18.48 ± 0.77 a			17.30 ± 2.20 b		12.95 ± 0.82 a		787 ± 42 a		836 ± 43 a
		pH3.6			54.51 ± 1.13 b		2.81 ± 0.28 a		0.16 ± 0.02 a			57.48 ± 1.21 b			18.25 ± 0.72 a			10.83 ± 1.32 a		11.99 ± 1.11 a		803 ± 52 a		844 ± 49 a
		pH3.8			51.36 ± 1.04 a		2.99 ± 0.19 a		0.37 ± 0.16 b			54.72 ± 0.96 a			18.41± 0.72 a			11.57 ± 1.16 a		12.60 ± 1.58 a		800 ± 42 a		842 ± 41 a
					***		n.s		***			***			n.s.			***		n.s.		n.s.		n.s.
Tan		Ctrl			52.35 ± 1.53 a		2.86 ± 0.11 a		0.43 ± 0.21 a			55.63 ± 1.42 a			18.59 ± 0.36 a			13.43 ± 1.71 a		13.43 ± 0.87 b		742 ± 17 a		787 ± 17 a
		TanA			54.15 ± 1.71 ab		2.92 ± 0.28 a		0.25 ± 0.09 a			57.32 ± 1.46 ab			18.12 ± 0.93 a			12.90 ± 2.68 a		12.41 ± 1.12 ab		827 ± 27 b		871 ± 26 b
		TanB			54.53 ± 1.84 b		2.92 ± 0.25 a		0.28 ± 0.15 a			57.73 ± 1.89 b			18.47 ± 0.73 a			13.21 ± 5.21 a		11.41 ± 0.86 a		821 ± 27 b		864 ± 26 b
					*		n.s.		n.s.			*			n.s.			n.s.		***		***		***
pH × Tan					***		**		***			***			n.s.			***		**		***		***
One-Way ANOVA																								
3.2		Ctrl			52.93 ± 0 cd		2.91 ± 0.17 abc		0.60± 0.05 e			56.44 ± 0.21 d			18.54 ± 0.46 a			15.60 ± 0.49 c		12.98 ± 0.88 bc		737 ± 10 a		785 ± 10 a
		TanA			55.28 ± 0.29 e		2.95 ± 0.08 abc		0.27± 0.01 bc			58.50 ± 0.22 ef			18.27 ± 2.04 a			16.23 ± 0.80 c		13.70 ± 0.29 bc		806 ± 32 bc		854 ± 32 bc
		TanB			55.634 ± 0.08 e		2.76 ± 0.19 a		0.49 ± 0.01 d			58.88 ± 0.10 f			18.53 ± 0.26 a			19.84 ± 1.46 d		12.55 ± 0.20 ab		821 ± 13 bc		872 ± 13 bc
3.6		Ctrl			53.16 ± 0.35 d		2.76 ± 0.12 ab		0.17 ± 0.02 a			56.10 ± 0.21 cd			18.80 ± 0.36 a			12.01 ± 0.29 b		13.03 ± 0.38 bc		741 ± 21 a		784 ± 21 a
		TanA			55.26 ± 0.68 e		2.63 ± 0.09 a		0.15 ± 0.02 a			58.04 ± 0.57 e			18.33 ± 1.04 a			10.55 ± 0.83 ab		11.33 ± 0.88 a		834 ± 13 c		874 ± 13 c
		TanB			55.41 ± 0.17 e		3.18 ± 0.14 bc		0.16 ± 0.03 a			58.75 ± 0.06 ef			17.70 ± 0.84 a			9.25 ± 1.06 a		10.96 ± 0.67 a		845 ± 27 c		883 ± 24 c
3.8		Ctrl			50.03 ± 0.56 a		2.89 ± 0.06 abc		0.56 ± 0.09 de			53.49 ± 0.53 a			18.38 ± 0.39 a			12.45 ± 0.29 b		14.48 ± 0.81 c		757 ± 17 ab		803 ± 17 ab
		TanA			51.90 ± 0.37 b		3.18 ± 0.26 c		0.34 ± 0.03 c			55.42 ± 0.08 bc			17.76 ± 0.94 a			11.92 ± 1.64 b		12.21 ± 0.66 ab		843 ± 35 c		884 ± 36 c
		TanB			52.15 ± 0.11 bc		2.89 ± 0.15 abc		0.22 ± 0 ab			55.26 ± 0.03 b			19.09 ± 0.29 a			10.33 ± 0.14 ab		11.09 ± 0.27 a		800 ± 26 abc		840 ± 26 abc
					***		**		***			***			n.s.			***		***		***		***
Two-Way ANOVA																								
pH	Tan			FERT (mg/L CAE)		COUT (mg/L CAE)		p-COUM (mg/L)			CAFT (mg/L CAE)			CA (mg/L)		Σ HXCA (mg/L)			RU (mg/L)		QUE-AGLC (mg/L)	QUE-GLC (mg/L)		Σ FLAVON (mg/L)
pH	pH3.2			2.78 ± 0.07 a		9.49 ± 0.07 b		0.22 ± 0.03 a			37.51 ± 0.20 a			1.39 ± 0.06 a		51.39 ± 0.33 b			2.99 ± 0.51 b		5.90 ± 0.38 a	25.19 ± 1.12 a		34.08 ± 1.28 ab
	pH3.6			2.77 ± 0.06 a		9.15 ± 0.19 a		0.19± 0.04 a			37.17 ± 0.47 a			1.50 ± 0.18 a		50.77 ± 0.56 ab			2.34 ± 0.41 a		6.00 ± 0.19 a	26.39 ± 1.30 a		34.73 ± 1.79 b
	pH3.8			2.61 ± 0.25 a		9.05 ± 0.09 a		0.17 ± 0.04 a			37.04 ± 0.53 a			1.56 ± 0.23 a		50.44 ± 0.71 a			2.08 ± 0.56 a		5.72 ± 0.23 a	25.24 ± 0.62 a		33.04 ± 0.95 a
				n.s.		***		n.s.			n.s.			n.s.		**			**		n.s.	*		*
Tan	Ctrl			2.70 ± 0.21 a		9.26 ± 0.21 a		0.19 ± 0.06 a			36.90 ± 0.43 a			1.46 ± 0.22 a		50.51 ± 0.74 a			3.08 ± 0.43 b		5.79 ± 0.28 a	26.66 ± 0.84 c		35.53 ± 1.10 c
	TanA			2.69 ± 0.20 a		9.26 ± 0.26 a		0.20 ± 0.03 a			37.33 ± 0.35 b			1.47 ± 0.14 a		50.94 ± 0.67 a			2.39 ± 0.44 a		5.78 ± 0.25 a	25.65 ± 0.65 b		33.83 ± 0.87 b
	TanB			2.76 ± 0.06 a		9.18 ± 0.23 a		0.20 ± 0.03 a			37.60 ± 0.21 b			1.52 ± 0.19 a		51.26 ± 0.33 a			1.93 ± 0.44 a		6.08 ± 0.25 a	24.41 ± 0.57 a		32.42 ± 0.49 a
				n.s.		n.s.		n.s.			***			n.s.		n.s.			***		n.s.	***		***
pH × Tan pH				n.s.		***		n.s.			*			n.s.		*			***		***	***		***
One-Way ANOVA																								
3.2	Ctrl			2.78 ± 0 a		9.45 ± 0.07 bc		0.22 ± 0.01 a			37.33 ± 0.16 d			1.38 ± 0.2 a		51.11 ± 0.27 bcd			3.58 ± 0.07 f		5.72 ± 0.02 abc	26.04 ± 0.09 d		35.33 ± 0.15 de
	TanA			2.79 ± 0.12 a		9.56 ± 0.01 c		0.22 ± 0.02 a			37.76 ± 0.02 ef			1.43 ± 0.05 a		51.75 ± 0.18 d			2.97 ± 0.01 e		5.60 ± 0.29 ab	25.78 ± 0.67 bcd		34.35 ± 0.98 cd
	TanB			2.73 ± 0 a		9.42 ± 0 bc		0.20 ± 0.07 a			37.43 ± 0.05 de			1.43 ± 0.01 a		51.22 ± 0.10 bcd			2.44 ± 0.07 c		6.38 ± 0.02 d	23.87 ± 0.25 a		32.69 ± 0.35 ab
3.6	Ctrl			2.76 ± 0.12 a		9.27 ± 0.02 abc		0.19 ± 0.09 a			36.87 ± 0.02 b			1.48 ± 0.08 a		50.57 ± 0.12 abc			2.83 ± 0.22 de		6.08 ± 0.38 cd	27.647 ± 0.39 e		36.55 ± 0.65 e
	TanA			2.78 ± 0.04 a		9.05 ± 0.33 a		0.18 ± 0.07 a			36.97 ± 0.1 bc			1.44 ± 0.05 a		50.42 ± 0.35 ab			2.07 ± 0.06 b		5.97 ± 0.27 bcd	26.09 ± 0.99 d		34.13 ± 1.32 bcd
	TanB			2.77 ± 0.02 a		9.07 ± 0.20 a		0.20 ± 0.01 a			37.83 ± 0.07 f			1.69 ± 0.33 a		51.55 ± 0.04 cd			1.97 ± 0.05 b		5.90 ± 0.15 bc	24.83 ± 0.54 abc		32.71 ± 0.75 ab
3.8	Ctrl			2.61 ± 0.44 a		8.98 ± 0.07 a		0.12 ± 0.02 a			36.35 ± 0.20 a			1.70 ± 0.35 a		49.76 ± 1.04 a			2.70 ± 0.02 cd		5.45 ± 0.02 a	25.96 ± 0.36 cd		34.11 ± 0.36 bcd
	TanA			2.51 ± 0.36 a		9.16 ± 0.0 ab		0.19 ± 0 a			37.26 ± 0.02 cd			1.54 ± 0.29 a		50.66 ± 0.64 abc			2.14 ± 0.03 b		5.77 ± 0.06 abc	25.10 ± 0.47 abcd		33.01 ± 0.50 abc
	TanB			2.73 ± 0.09 a		9.02 ± 0.01 a		0.21 ± 0.01 a			37.51 ± 0.04 def			1.44 ± 0.01 a		50.90 ± 0.08 bcd			1.41 ± 0.01 a		5.94 ± 0.15 bcd	24.65 ± 0.09 ab		31.99 ± 0.26 a
				n.s.		***		n.s.			***			n.s.		***			***		***	***		***
Two-Way ANOVA																								
pH			Tan				Dp-3-*O*-glc (mg/L MAE)			Cy-3-*O*-glc (mg/L MAE)			Pt-3-*O*-glc (mg/L MAE)				Pn-3-*O*-glc (mg/L MAE)			Mv-3-*O*-glc (mg/L)			Σ ANTHOCYANINS	
pH			pH3.2				7.74 ± 0.16 a			1.60 ± 0.09 b			11.29 ± 0.46 b				4.24 ± 0.18 b			90.74 ± 2.93 a			116 ± 1.44 a	
			pH3.6				7.44 ± 0.32 a			1.49 ± 0.12 ab			10.56 ± 0.35 a				4.01 ± 0.11 a			88.16 ± 3.40 a			112 ± 1.44 a	
			pH3.8				7.54 ± 0.42 a			1.46 ± 0.10 a			10.77 ± 0.52 ab				4.01 ± 0.15 a			88.55 ± 2.23 a			112 ± 2.92 a	
							n.s.			*			**				**			n.s.			n.s.	
Tan			Ctrl				7.79 ± 0.33 a			1.62 ± 0.05 c			11.23 ± 0.33 b				4.26 ± 0.19 b			91.58 ± 2.03 b			116 ± 2.17 b	
			TanA				7.43 ± 0.36 a			1.51 ± 0.11 b			10.96 ± 0.56 b				4.05 ± 0.13 a			89.61 ± 2.39 b			114 ± 2.17 b	
			TanB				7.49 ± 0.17 a			1.41 ± 0.07 a			10.39 ± 0.34 a				3.96 ± 0.12 a			86.29 ± 2.41 a			110 ± 2.50 a	
							n.s.			***			**				***			***			***	
pH × Tan							**			***			***				***			**			***	
One-way ANOVA																								
3.2			Ctrl				7.77 ± 0.17 abc			1.64 ± 0.02 e			11.49 ± 0.03 c				4.46 ± 0.03 c			93.30 ± 0.68 c			119 ± 0.77 d	
			TanA				7.85 ± 0.10 bc			1.65 ± 0.03 e			11.64 ± 0.10 c				4.18 ± 0 b			91.57 ± 0.48 bc			117 ± 0.77 cd	
			TanB				7.64 ± 0.10 abc			1.47 ± 0.07 bcd			10.57 ± 0.34 a				4.11 ± 0.10 ab			88.30 ± 2.53 abc			112 ± 3.01 abc	
3.6			Ctrl				7.59 ± 0.51 abc			1.60 ± 0.03 de			10.83 ± 0.24 ab				4.09 ± 0.10 ab			90.94 ± 2.40 abc			115 ± 1.58 bcd	
			TanA				7.20 ± 0.14 a			1.47 ± 0.03 bc			10.50 ± 0.50 a				3.96 ± 0.10 ab			87.23 ± 1.85 ab			110 ± 0.77 ab	
			TanB				7.54 ± 0.17 abc			1.34 ± 0.03 a			10.23 ± 0.21 a				3.94 ± 0.06 ab			85.47 ± 2.60 a			109 ± 2.14 a	
3.8			Ctrl				8.07 ± 0.21 c			1.59 ± 0.03 cde			11.35 ± 0.27 bc				4.15 ± 0.17 b			89.56 ± 1.00 abc			115 ± 1.69 bcd	
			TanA				7.25 ± 0.14 ab			1.41 ± 0 ab			10.73 ± 0 ab				4.01 ± 0.17 ab			90.04 ± 1.20 abc			113 ± 1.51 abcd	
			TanB				7.31 ± 0.03 ab			1.39 ± 0.04 ab			10.22 ± 0.14 a				3.86 ± 0 a			86.05 ± 0.41 ab			109 ± 0.55 a	
							**			***			***				***			**			***	

**Table 5:** Mean separation of color features (one-way and two-way ANOVA) of Sangiovese red wines obtained under different pH and Tan conditions, along with the interactive effect of both factors. Letters next to the values indicate groups based on Tukey HSD comparison tests. Signification codes: *** *p*-value < 0.001; ** *p*-value < 0.01; * *p*-value < 0.05; *p*-value < 0.1; n.s. not significant.

Two-Way ANOVA										
pH	***Tan***	L*	a*	b*	C*	H* (°)	FA (%)	Co (%)	PP (%)	LPP/PP (%)
pH	pH3.2	15.88 ± 1.90 a	46.75 ± 2.66 b	26.21 ± 3.1 b	53.62 ± 3.82 b	29.17 ± 1.63 a	41.5 ± 1.9 a	28.4 ± 1.8 b	30.1 ± 0.8 a	51.3 ± 5.9 a
	pH3.6	14.54 ± 1.05 a	43.22 ± 1.40 a	23.15 ± 1.63 a	49.04 ± 2.00 a	28.15 ± 0.93 a	43.8 ± 2.7 a	26.1 ± 2.1 a	30.1 ± 1.0 a	52.1 ± 1.8 a
	pH3.8	14.40 ± 1.20 a	41.48 ± 1.48 a	22.13 ± 1.79 a	47.02 ± 2.14 a	28.03 ± 1.10 a	41.9 ± 2.6 a	27.6 ± 1.2 ab	30.5 ± 1.8 a	52.3 ± 1.9 a
		n.s.	***	**	***	n.s.	n.s.	*	n.s.	n.s.
Tan	Ctrl	15.20 ± 2.12 a	44.34 ± 4.09 a	24.27 ± 4.03 a	50.57 ± 5.53 a	28.55 ± 1.77 a	45.4 ± 1.7 b	25.5 ± 1.7 a	29.1 ± 1.0 a	51.6 ± 2.5 a
	TanA	15.13 ± 1.29 a	44.18 ± 2.96 a	24.29 ± 2.58 a	50.42 ± 3.83 a	28.73 ± 1.03 a	41.5 ± 1.1 a	28.6 ± 1.3 b	29.9 ± 0.5 a	50.8 ± 5.4 a
	TanB	14.62 ± 1.26 a	43.33 ± 1.56 a	23.26 ± 1.76 a	49.19 ± 2.14 a	28.18 ± 1.18 a	40.4 ± 1.1 a	28.2 ± 1.0 b	31.5 ± 0.9 b	53.1 ± 1.7 a
		n.s.	n.s	n.s	n.s	n.s	***	***	***	n.s.
pH × Tan		**	***	***	***	**	***	***	***	n.s.
One-Way ANOVA										
3.2	Ctrl	17.53 ± 0.75 b	49.03 ± 0.88 d	28.86 ± 1.31 c	56.89 ± 1.43 d	30.47 ± 0.68 b	44.0 ± 0.0 d	26.3 ± 0.5 b	29.7 ± 0.5 ab	51.9 ± 4.8 a
	TanA	16.27 ± 0.51 ab	47.33 ± 0.70 cd	26.89 ± 0.85 bc	54.44 ± 1.02 cd	29.60 ± 0.41 ab	40.3 ± 0.2 ab	30.1 ± 0.4 d	29.6 ± 0. 6 ab	48.0 ± 11.4 a
	TanB	13.85 ± 1.75 a	43.89 ± 2.54 abc	22.89 ± 2.89 ab	49.52 ± 3.59 abc	27.43 ± 1.61 a	40.3 ± 0.2 ab	28.8 ± 0.9 cd	30.9 ± 1.1 bc	54.1± 0.6 a
3.6	Ctrl	13.30 ± 0.75 a	41.43 ± 0.88 ab	21.23 ± 1.31 a	46.55 ± 1.43 ab	27.13 ± 1.61 a	46.8 ± 0 e	23.4 ± 0.3 a	29.8 ± 0.3 ab	51.3 ± 0.6 a
	TanA	15.53 ± 0.51 ab	44.55 ± 0.70 bc	24.69 ± 0.85 abc	50.93 ± 1.02 bc	29.00 ± 0.41 ab	42.4 ± 0.1 c	27.7 ± 0.6 bc	29.9 ± 0.5 ab	52.6 ± 0.3 a
	TanB	14.63 ± 1.75 a	43.41 ± 2.54 ab	23.29 ± 2.89 ab	49.26 ± 3.59 abc	28.20 ± 1.61 ab	41.7 ± 0 bc	27.2 ± 0.8 bc	31.1 ± 0.8 bc	53.2 ± 0.5 a
3.8	Ctrl	13.67 ± 1.16 a	40.53 ± 1.52 a	20.81 ± 1.71 a	45.57 ± 2.13 a	27.15 ± 1.03 a	45.0 ± 0.8 d	26.5 ± 2.3 bc	28.5 ± 1.6 a	52.6 ± 1.5 a
	TanA	13.60 ± 0.82 a	40.67 ± 0.96 a	21.28 ± 1.35 a	45.91 ± 1.48 ab	27.60 ± 0.94 a	41.8 ± 0.9 c	27.9 ± 0.1 bcd	30.3 ± 0.9 ab	51.7 ± 0.9 a
	TanB	15.37 ± 0.84 ab	42.71 ± 1.02 ab	23.59 ± 1.19 ab	48.8 ± 1.47 ab	28.90 ± 0.66 ab	39.1 ± 0.3 a	28.5 ± 0.4 bcd	32.3 ± 0.7 c	52.1 ± 3.3 a
		***	***	***	***	**	***	***	***	n.s.

**Table 6:** Results from the color stability test. Since the interaction between the factors pH × Tan was not significant (*p*-value > 0.1), the results are presented independently for each factor. Letters next to the values indicate groups based on Tukey HSD comparison tests for each level within each factor. The significance of individual factors (*p* ≤ 0.05) is shown below each summary table.

pH	Cold Stability (ΔE*)
3.2	2.20 ± 1.28 a
3.6	2.45 ± 1.19 a
3.8	6.56 ± 3.04 b
Significance of factor	***
**Tan**	Cold Stability (ΔE*)
CTRL	4.12 ± 3.00 a
TAN A	4.16 ± 3.49 a
TAN B	2.93 ± 1.85 a
Significance of factor	n.s.

Signification codes: *** *p*-value < 0.001; ** *p*-value < 0.01; * *p*-value < 0.05; *p*-value < 0.1; n.s. not significant.

## Data Availability

The metadata will be made available and searchable on the AMS Acta repository (https://amsacta.unibo.it/) by recording the DOI of the article soon after publishing.

## Supplementary Materials

The following supporting information can be downloaded at: https://www.mdpi.com/article/10.3390/foods15122161/s1, Figure S1: MALDI-TOF MS spectrum (linear positive mode) of the grape extract labeled TanA; Figure S2: Details of the MALDI-TOF MS spectrum (linear positive mode) of the grape extract labeled TanB. Signals 200–2500 *m*/*z*; Figure S3: PC1-PC2 biplot of polyphenolic compounds according to the different treatments; Figure S4: Refined PCA biplot. Table S1: HPLC-DAD method calibration and validation; Table S2: PCA correlation matrix: Squared cosines of the variables.

## Abbreviations

The following abbreviations are used in this manuscript:

FA	Free anthocyanins
Co	Copigmentation
PP	Polymeric pigment
BSA	Bovine serum albumin
LPP	Large polymeric pigments
TPC	Total polyphenolic content
PAs	Proanthocyanidins
GA	Gallic acid
PROT	Protocatechuic acid
SA	Syringic acid
Σ HBA	Total hydroxybenzoic acids
(+)-CAT	(+)-Catechin
(-)-EPI	(-)-Epicatechin
PRO B2	Procyanidin B2
Σ FLAVAN	Total flavan-3-ols
CE	(+)-CAT equivalents
FERT	Fertaric acid
COUT	Coutaric acid
CAFT	Caftaric acid
CA	Caffeic acid
p-COUM	p-Coumaric acid
CAE	Caffeic acid equivalents
Σ HXCA	Total hydroxycinnamic acids
RU	Rutin
QUE-AGLC	Quercetin aglycone
QUE-GLC	Quercetin glycoside
Σ FLAVON	Total flavonols
Dp-3-*O*-glc	Delphinidin-3-O- glycoside
Cy-3-*O*-glc	Cyanidin-3-O- glycoside
Pt-3-*O*-glc	Petunidin-3-O- glycoside
Pn-3-*O*-glc	Peonidin-3-*O*-glc
Mv-3-*O*-glc	Malvidin-3-*O*-glc
Σ ANTHOCYANINS	total free anthocyanins (HPLC)
MAE	Mv-3-*O*-glc equivalents
AH assay	Adams-Harbertson colorimetric assay [26]
Ctrl	Control wines (no tannin added)
Peak assignment MALDI-TOF MS	C: Catechins; G: Gallocatechins; CG: Catechin gallate; GCG: Gallocatechin gallate.
