# Supplementary material for: Precision Enology Strategies to Enhance the Quality of Red Wine Color: The Synergistic Effect of pH and Selected Exogenous Grape Seed Tannins
Source: Foods. 2026 Jun 15;15(12):2161. doi: 10.3390/foods15122161 (PMC13297818; doi:10.3390/foods15122161)
Supplement: Supplementary file 1 [file foods-15-02161-s001.zip › Supplementary Figure S1.pdf]

Supplementary Figure S1

MALDI-TOF MS spectrum (linear positive mode) of the grape extract labelled TanA. Signals were confined to the region 200 – 600 m/z.

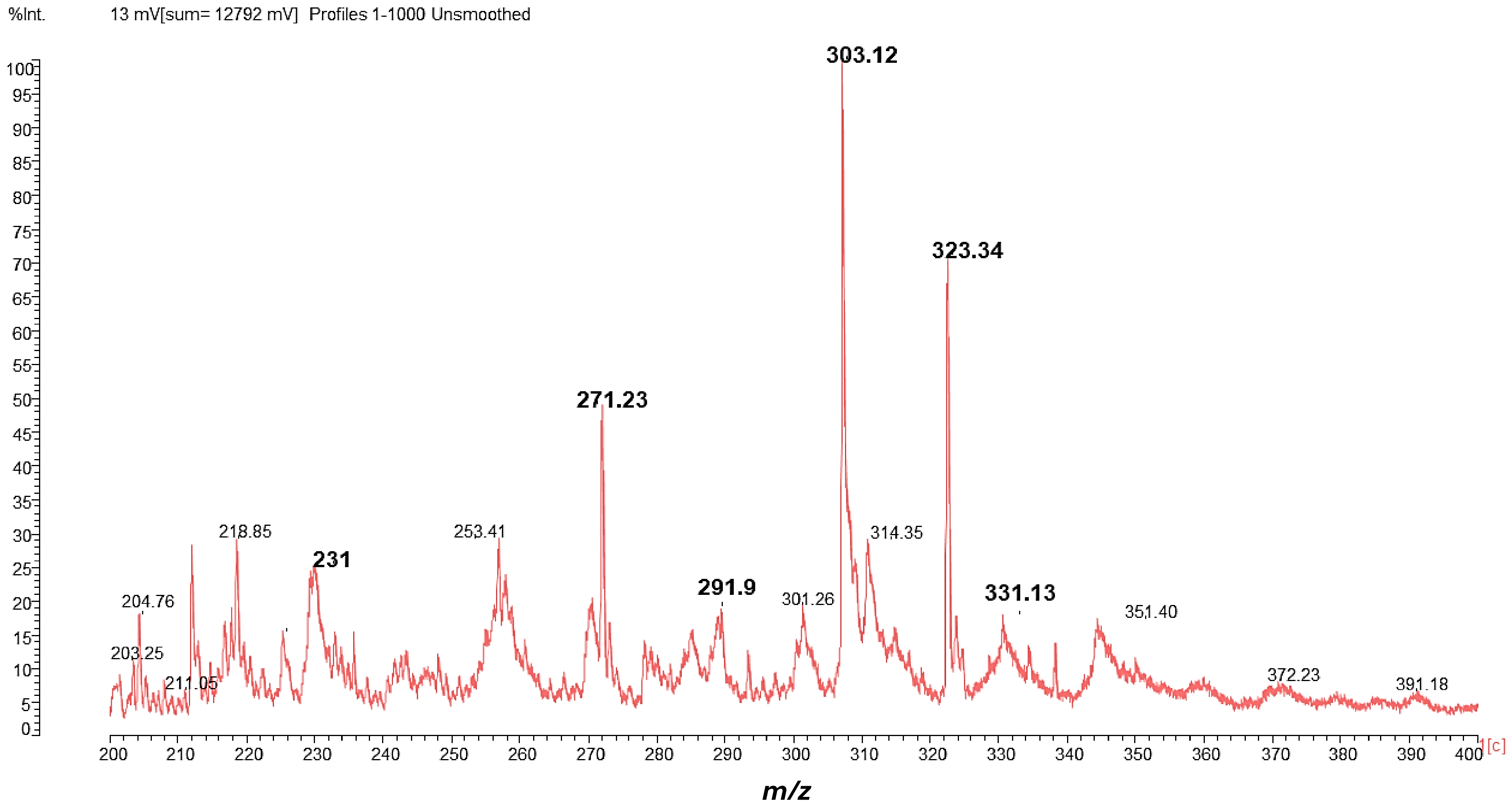

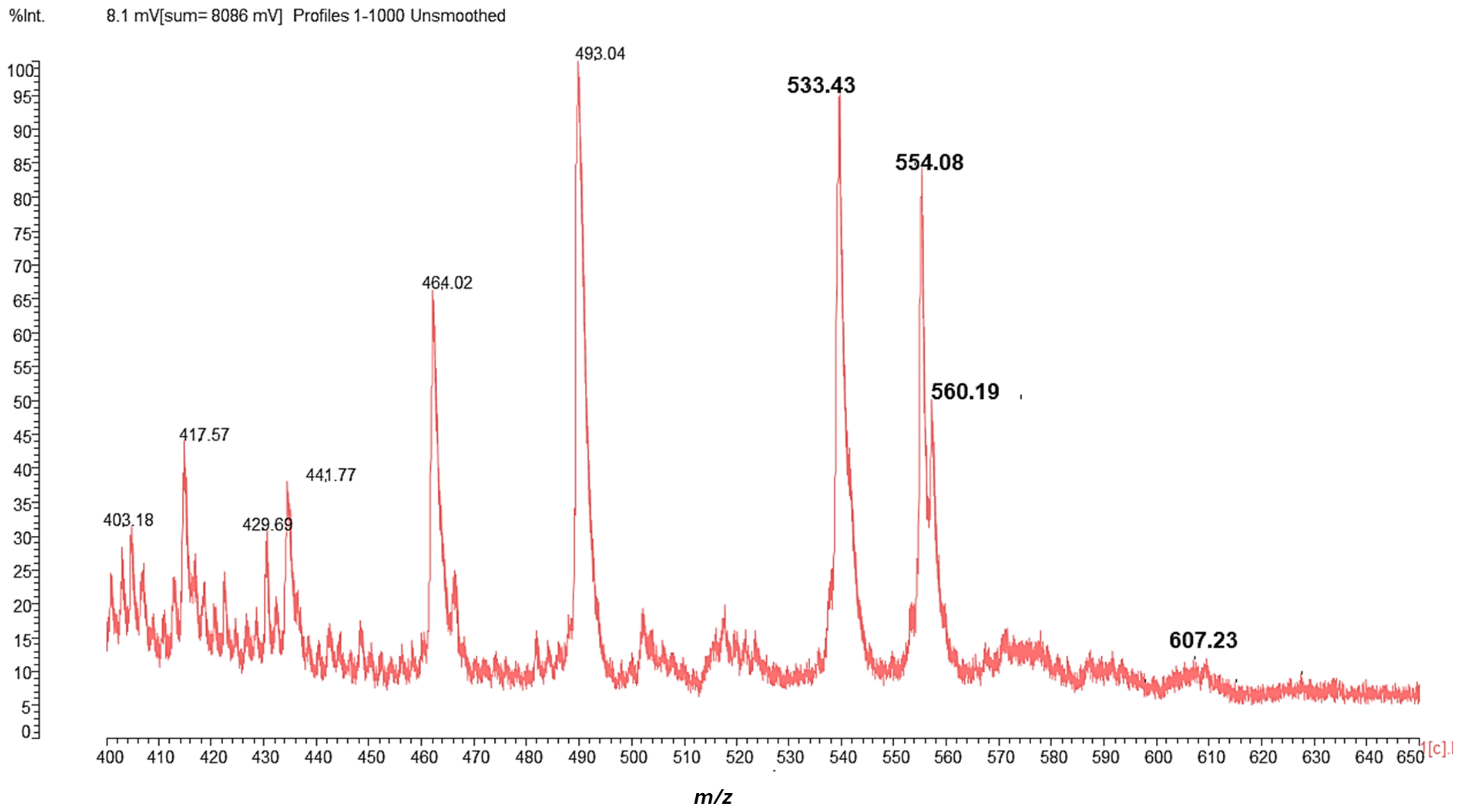
%Int.
8.1 mV[sum= 8086 mV] Profiles 1-1000 Unsmoothed
100
95
90
85
80
75
70
65
60
55
50
45
40
35
30
25
20
15
10
5
0
493.04
533.43
554.08
464.02
560.19
417.57
441.77
403.18
429.69
607.23
400
410
420
430
440
450
460
470
480
490
500
510
520
530
540
550
560
570
580
590
600
610
620
630
640
650
1[c].I
m/z
