# Supplementary material for: Precision Enology Strategies to Enhance the Quality of Red Wine Color: The Synergistic Effect of pH and Selected Exogenous Grape Seed Tannins
Source: Foods. 2026 Jun 15;15(12):2161. doi: 10.3390/foods15122161 (PMC13297818; doi:10.3390/foods15122161)
Supplement: Supplementary file 1 [file foods-15-02161-s001.zip › Supplementary Figure S2.pdf]

Supplementary Figure S2

Details of the MALDI-TOF MS spectrum (linear positive mode) of the grape extract labelled TanB. Signals 200 – 2500 m/z. The assignment of relevant peaks was reported in **Table 2** of the manuscript.

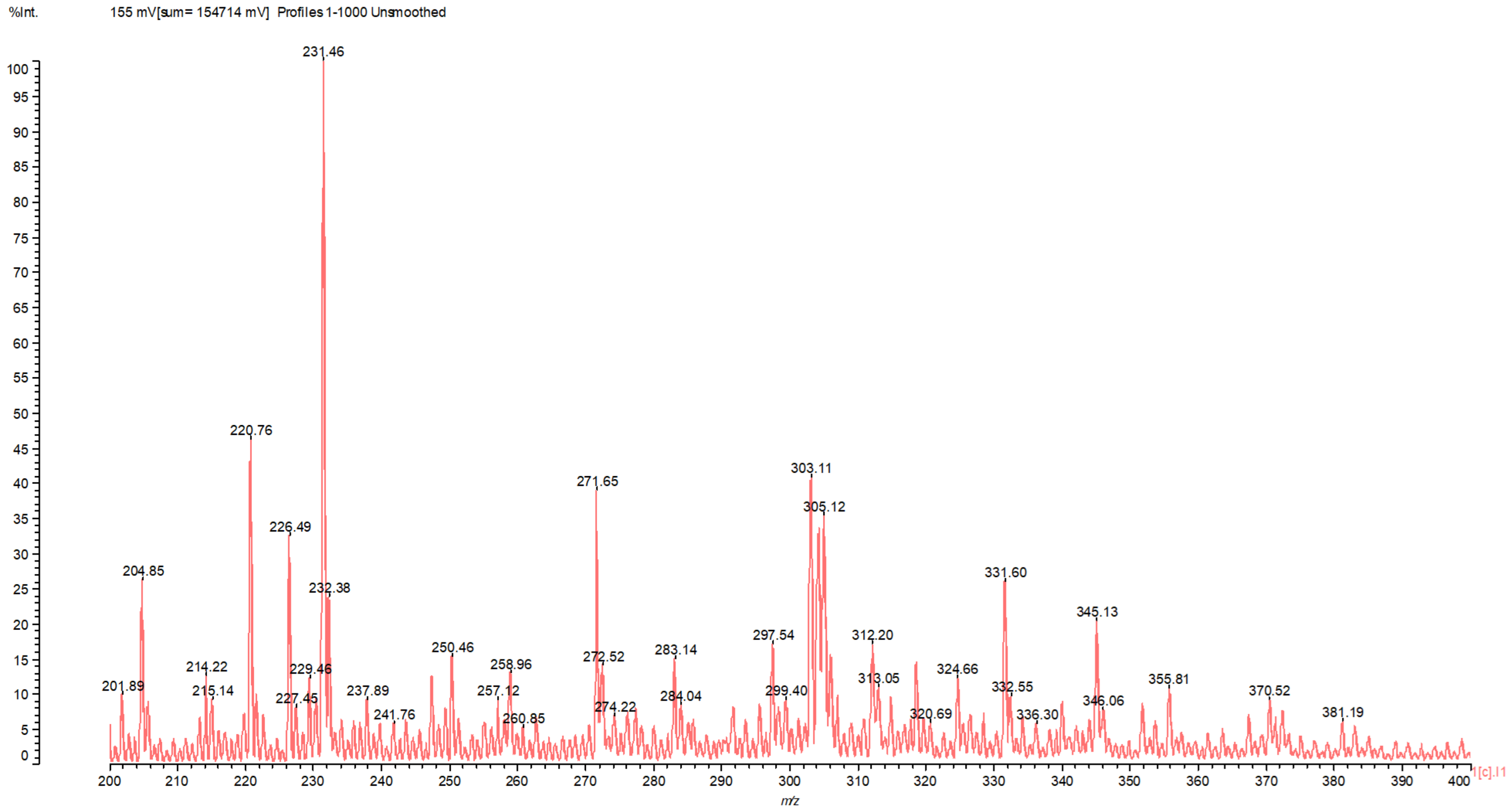

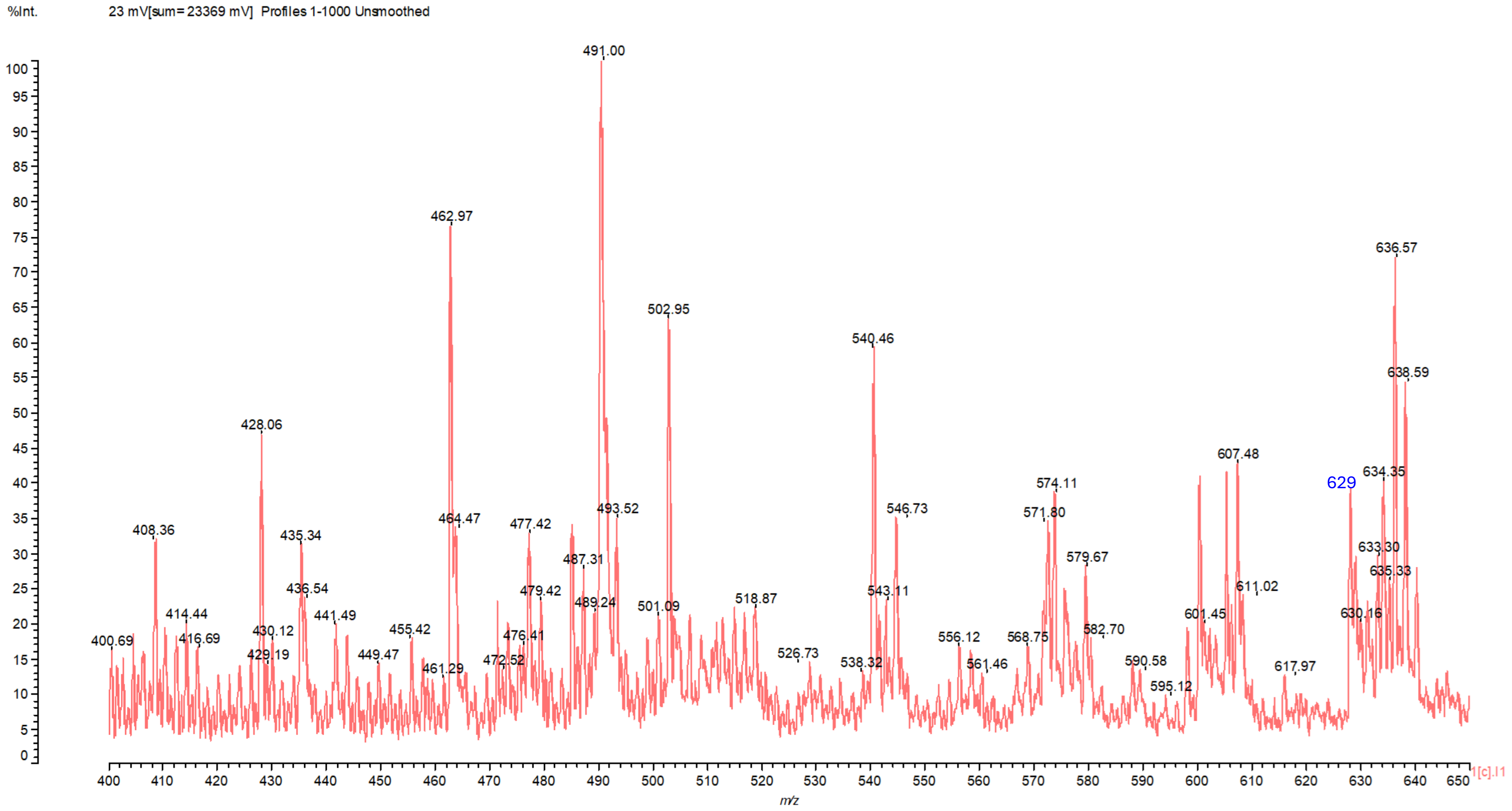
%Int.
23 mV[sum= 23369 mV] Profiles 1-1000 Unsmoothed
491.00
462.97
636.57
502.95
540.46
638.59
428.06
607.48
629
574.11
634.35
493.52
546.73
464.47
571.80
408.36
477.42
435.34
633.30
487.31
579.67
635.33
436.54
479.42
543.11
611.02
489.24
501.09
518.87
414.44
441.49
601.45
630.16
430.12
455.42
582.70
400.69
416.69
476.41
556.12
568.75
429.19
449.47
526.73
472.52
538.32
561.46
590.58
617.97
461.29
595.12
m/z
1[c].I1

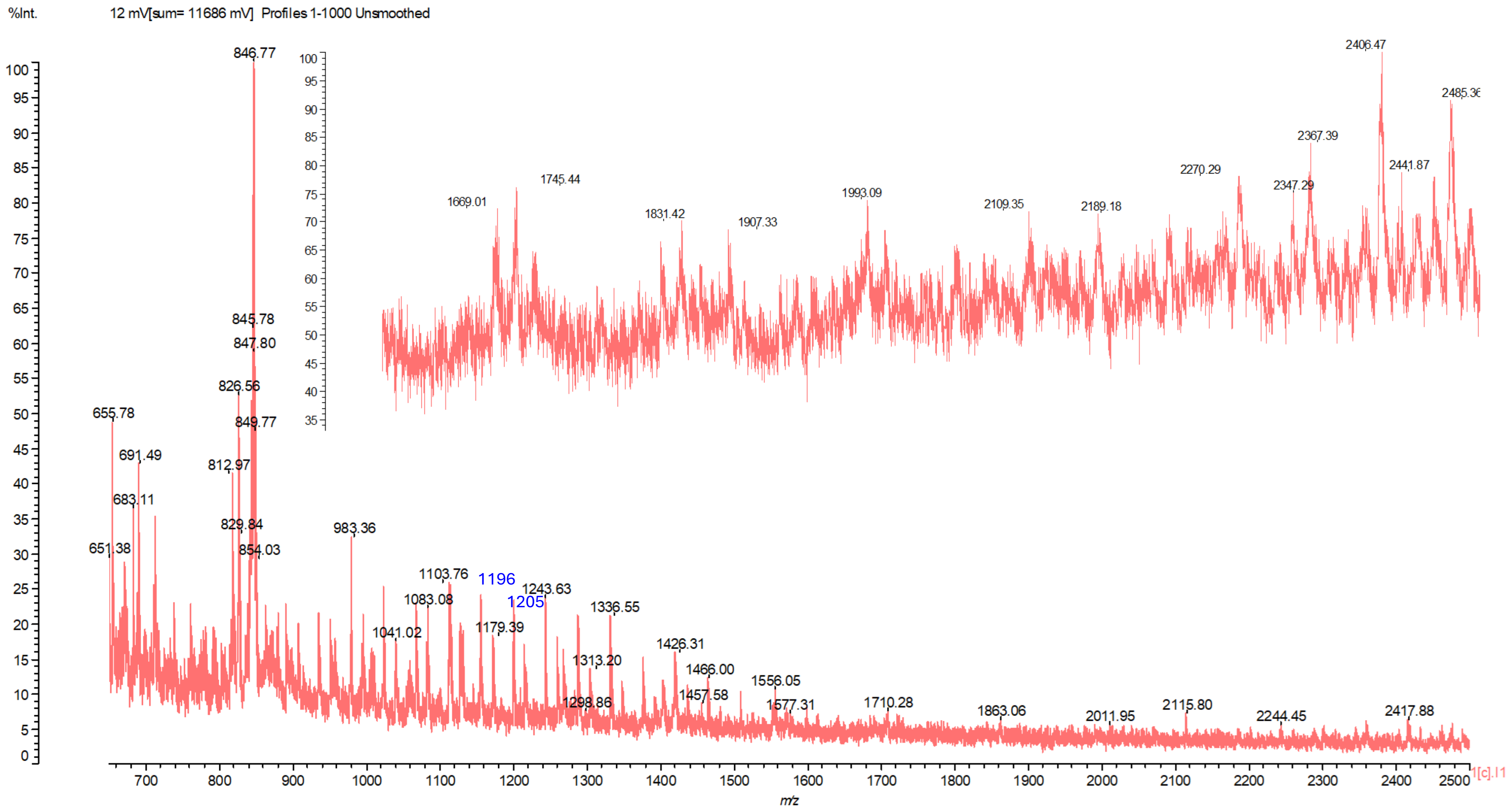
%Int.
12 mV[sum= 11686 mV] Profiles 1-1000 Unsmoothed
846.77
845.78
847.80
826.56
655.78
849.77
691.49
812.97
683.11
829.84
651.38
854.03
983.36
1103.76
1196
1243.63
1083.08
1205
1336.55
1041.02
1179.39
1426.31
1313.20
1466.00
1556.05
1457.58
1298.86
1577.31
1710.28
1863.06
2011.95
2115.80
2244.45
2417.88
1669.01
1745.44
1831.42
1907.33
1993.09
2109.35
2189.18
2270.29
2347.29
2367.39
2406.47
2441.87
2485.36
m/z
1[c].I1
