# Supplementary material for: Precision Enology Strategies to Enhance the Quality of Red Wine Color: The Synergistic Effect of pH and Selected Exogenous Grape Seed Tannins
Source: Foods. 2026 Jun 15;15(12):2161. doi: 10.3390/foods15122161 (PMC13297818; doi:10.3390/foods15122161)

Supplementary Figure S3

PC1-PC2 biplot of polyphenolic compounds according to the different treatments.

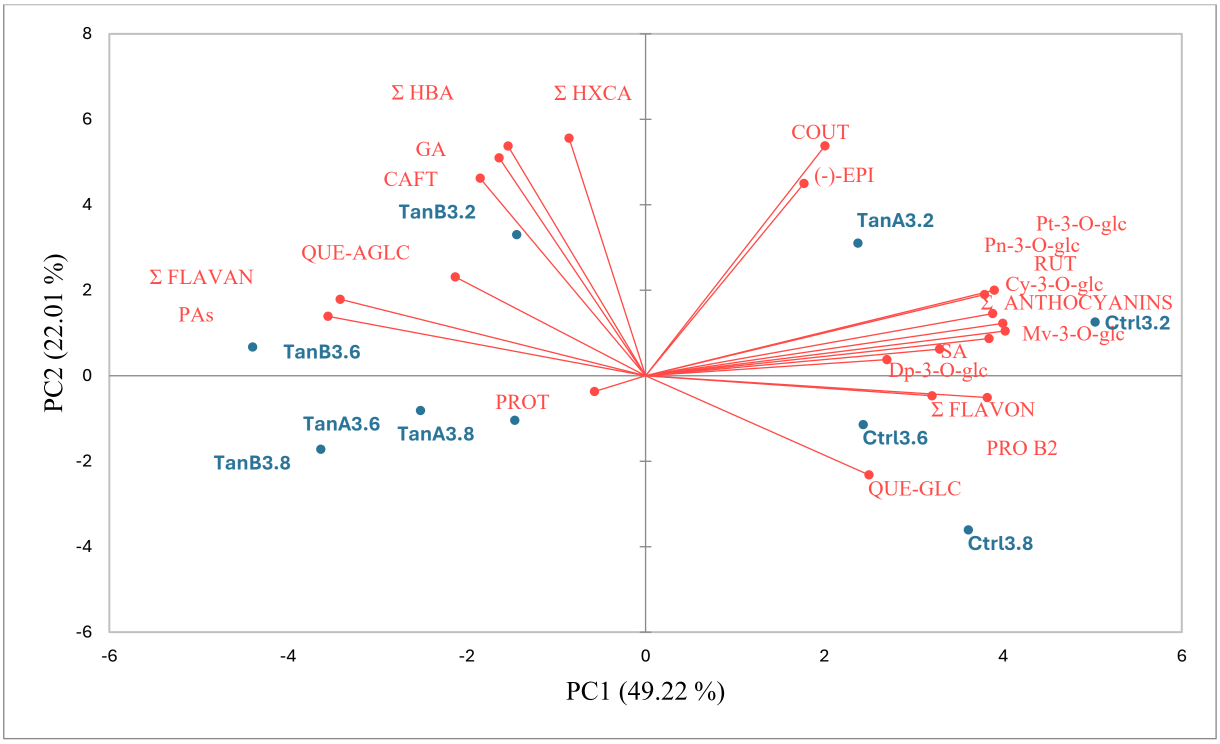

Supplement: Supplementary file 1 [file foods-15-02161-s001.zip › Supplementary Figure S3.pdf]
