# Supplementary material for: Precision Enology Strategies to Enhance the Quality of Red Wine Color: The Synergistic Effect of pH and Selected Exogenous Grape Seed Tannins
Source: Foods. 2026 Jun 15;15(12):2161. doi: 10.3390/foods15122161 (PMC13297818; doi:10.3390/foods15122161)

Supplementary Figure S4.

Refined PCA Biplot including variables with the highest squared cosines (> 0.5, supplementary Table S2) for the first two dimensions (PC1 and PC2).

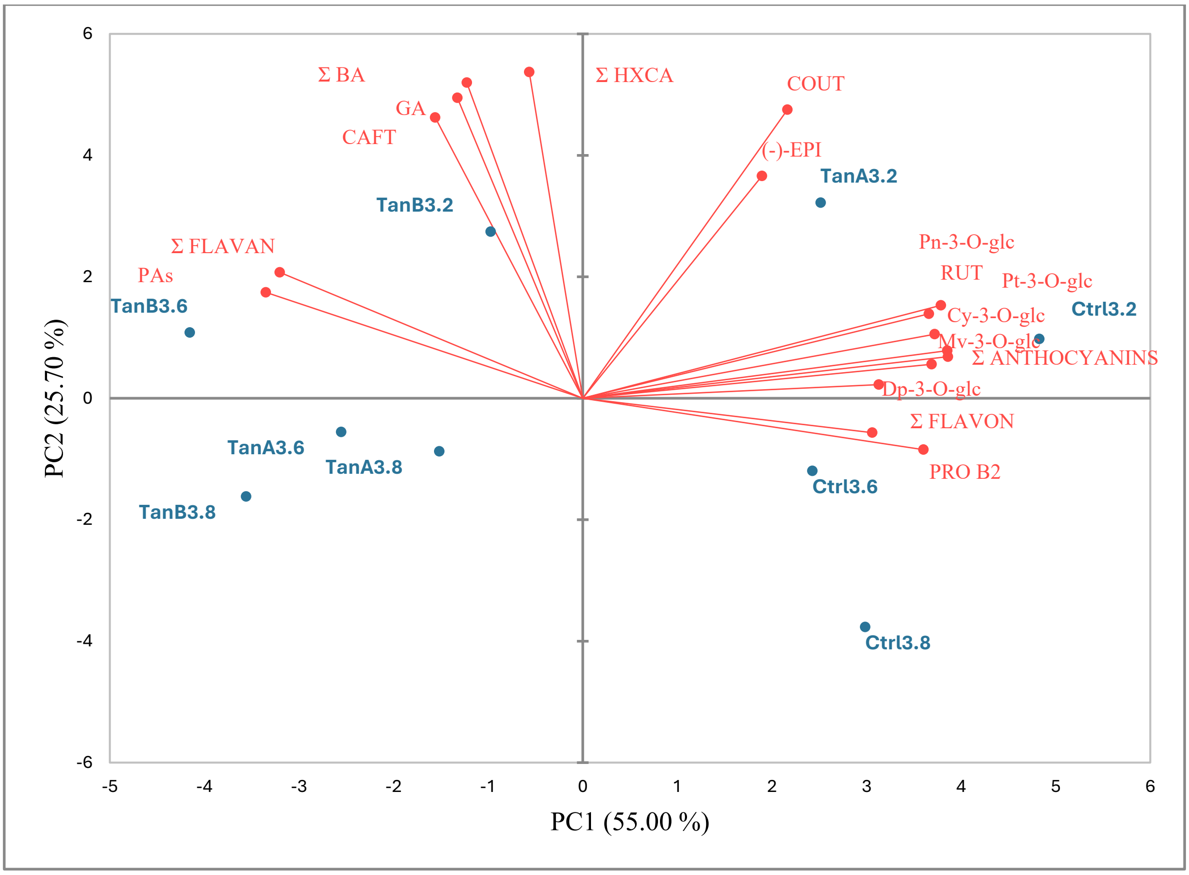

Supplement: Supplementary file 1 [file foods-15-02161-s001.zip › Supplementary Figure S4.pdf]
