# Supplementary material for: Precision Enology Strategies to Enhance the Quality of Red Wine Color: The Synergistic Effect of pH and Selected Exogenous Grape Seed Tannins
Source: Foods. 2026 Jun 15;15(12):2161. doi: 10.3390/foods15122161 (PMC13297818; doi:10.3390/foods15122161)
Supplement: Supplementary file 1 [file foods-15-02161-s001.zip › Supplementary Table S1.pdf]

Supplementary Table S1 – HPLC-DAD method calibration and validation

Quantification of HPLC-DAD was made using calibration curves obtained by injecting known amounts of pure compounds as external standards (calibration ranges: 0 – 50 mg/L); these are reported in **Table S1**, along with the validation parameters. The limits of detection (LOD) were calculated considering a signal-to-noise ratio (*S*/*N*) of 3 and the limits of quantification (LOQ) were calculated considering a signal-to-noise ratio (*S*/*N*) of 10 for every standard, and the baseline noise was measured considering a peak-to-peak measurement within 3 min in three distinguished sections of the chromatogram.

Recovery performances were calculated as percent ratio between the concentrations observed/expected, injecting a red wine spiked with three concentration levels (1, 2, 4 mg/L) of each standard. The recovery values typically ranged 101-94% with relative standard deviation RSD always lower than 5%.

The intra-day and inter-day repeatability were estimated for the standard concentration by analyzing the same wine sample three times a day and for 3 consecutive days. The intra-day and inter-day relative standard deviations RSD for the concentrations were generally better than 6%.

Table S1. Performances of the calibration curves of individual phenolic reference standards. Regression parameters averaged over three calibrations replicates.

	**Wavelenght of detection**	**Regression equation y = ax + b**			**$LOD_{(S/N=3)}$**	**$LOQ_{(S/N=10)}$**
Compound	λ (nm)	a	b	r2	(µg/L)	(µg/L)
GA	280	8.81 ± 0.001	0.09± 0.01	0.999	121	151
PROT	256	11.04 ± 0.02	0.05± 0.02	0.999	153	160
SA	280	9.91± 0.02	0.12± 0.01	0.998	250	250
(+)-CAT	280	2.18± 0.001	0.32± 0.003	0.998	262	358
(-)-EPI	280	2.46± 0.001	0.71± 0.02	0.999	350	652
PRO B2	280	3.33± 0.04	0.32± 0.002	0.999	210	1180
p-COUM	308	19.5± 0.03	0.67± 0.031	0.998	680	868
CA	324	11.76± 0.04	0.52± 0.07	0.998	1100	1810
RU	356	3.77± 0.001	0.08± 0.03	0.998	1212	1822
QUE-AGLC	356	20.51± 0.12	0.16± 0.01	0.998	885	1772
QUE-GLC	356	17.51± 0.05	0.18± 0.02	0.999	604	1096
Mv-3-*O*-glc	520	3.57± 0.61	0.06± 0.004	0.998	1120	2544
