# Supplementary material for: Precision Enology Strategies to Enhance the Quality of Red Wine Color: The Synergistic Effect of pH and Selected Exogenous Grape Seed Tannins
Source: Foods. 2026 Jun 15;15(12):2161. doi: 10.3390/foods15122161 (PMC13297818; doi:10.3390/foods15122161)
Supplement: Supplementary file 1 [file foods-15-02161-s001.zip › Supplementary Table S2.pdf]

Supplementary Table S2. PCA correlation matrix: Squared cosines of the variables.

POLYPHENOLIC COMPOUNDS	PC1	PC2
GA	0.149	**0.645**
PROT	0.018	0.003
SA	0.404	0.004
Σ HBA	0.131	**0.716**
(-)-EPI	0.174	**0.502**
PRO B2	**0.811**	0.006
PAs	**0.700**	0.048
Σ FLAVAN	**0.647**	0.079
COUT	0.223	**0.717**
CAFT	0.190	**0.529**
Σ HXCA	0.041	**0.766**
RUT	**0.844**	0.099
QUE-AGLC	0.251	0.133
QUE-GLC	0.346	0.134
Σ FLAVON	**0.570**	0.005
Dp-3-O-glc	**0.600**	0.010
Cy-3-O-glc	**0.886**	0.037
Pt-3-O-glc)	**0.836**	0.052
Pn-3-O-glc	**0.797**	0.089
Mv-3-O-glc	**0.819**	0.019
Σ ANTHOCYANINS	**0.898**	0.027
